# A computational framework for identifying cytoskeletal genes associated with age-related diseases

**DOI:** 10.1038/s41598-025-97363-y

**Published:** 2025-04-26

**Authors:** Reem A. Elghaish, Nayera E. Attallah, Hesham Khaled, Asmaa S. Mekawy, Menattallah Elserafy, Eman Badr

**Affiliations:** 1 University of Science and Technology, Zewail City of Science and Technology, Giza, 12578 Egypt; 2 Center for Genomics, Helmy Institute for Medical Sciences, Zewail City of Science and Technology, Giza, 12578 Egypt; 3 Faculty of Computers and Artificial Intelligence, Cairo University, Giza, 12613 Egypt

**Keywords:** Age-related Diseases, Cytoskeleton, Machine learning, Differential expression analysis, Biomarkers, Diseases, Computational models

## Abstract

The cytoskeleton comprises polymers from protein filaments shaped in an organized structure. This structure contributes significantly to the cell’s function and viability. Decades of research have implicated that the cytoskeleton’s dynamic nature is associated with downstream signaling events that further regulate cellular activity and control aging and neurodegeneration. This study aims to investigate the transcriptional changes of the cytoskeletal genes and their regulators in five age-related diseases: Hypertrophic Cardiomyopathy (HCM), Coronary Artery Disease (CAD), Alzheimer’s disease (AD), Idiopathic Dilated Cardiomyopathy (IDCM), and Type 2 Diabetes Mellitus (T2DM). An integrative approach of machine learning-based models and differential expression analysis was employed to identify potential biomarkers based on the cytoskeletal genes. Multiple machine-learning algorithms were used, where the Support Vector machines (SVM) classifier achieved the highest accuracy. The study highlighted 17 genes involved in the cytoskeleton’s structure and regulation associated with age-related diseases. The results provide a holistic overview of the role of transcriptionally dysregulated cytoskeletal genes in age-related diseases. This study pinpoints cytoskeletal genes and regulators of the cytoskeleton that can be utilized as potential markers and drug targets.

## Introduction

Aging is an irreversible gradual process that leads to a progressive decline in cells’ function^1^. For a long time, scientists have proposed an association between aging and multiple chronic disorders in humans. For example, aging escalates the risk of numerous common diseases such as Alzheimer’s disease (AD)^2^, cardiovascular diseases^3^, diabetes^4^, and pulmonary disease (COPD)^5^. In addition, after the age of 60, many elderly patients have numerous comorbidities with increasing age. Meanwhile, aging is considered a leading risk factor for most chronic diseases, yet many research efforts are still needed to identify therapeutic targets that promote healthy aging and longevity.

Cytoskeleton is a network of intracellular filamentous proteins that are involved in a plethora of functions. These proteins are in constant flux and connect the cell to its external environment, supporting the proper spatial organization of cells’ contents. They also maintain cellular shape and integrity and generate forces to promote cellular motility^6–9^. The cell’s cytoskeleton comprises three polymers: microfilaments (actin filaments), intermediate filaments, and microtubules^6^. Cytoskeletal integrity is essential to various cellular processes, such as intracellular trafficking and phagocytosis^10^. Considering the essential role of the cytoskeleton in enhancing cellular integrity, it is no surprise that any alteration in its dynamics or organization results in diseases ranging from cancer to neurodegeneration^11^. However, despite the cytoskeleton’s critical role in the homeostasis and functions of neurons, little is known about its participation in the physiological development of aging and neurodegeneration.

Several studies reported that defects in the cytoskeleton result in myopathies through altered signaling and structural mechanisms. Those defects can be categorized based on location: the plasma membrane, sarcomere proteins, and the inner nuclear membrane^12^. For example, in IDCM patients, the expression of sarcomeric and cytoskeletal proteins was altered, especially the decreased expression of the α-myosin heavy chain and β-actin^13^. In addition, several mutations in genes encoding sarcomere or sarcomere-associated proteins were reported in HCM. Studies also linked nine genes: beta myosin heavy chain, myosin binding protein C, troponin T, troponin I, troponin C, alpha tropomyosin, actin, regulatory light chain, and essential light chain to HCM^14,15^. Moreover, multiple genetic variants in genes involved in cytoskeletal assembly regulation, such as SPTBN5, ADAMTS7, and NID2, have been identified in CAD patients^16^.

Owing to the cytoskeleton’s essential role in cell transport and the normal functioning of neurons, cytoskeletal aberrations lead to damage of neurons and cell death, which are the common drivers for neurodegenerative diseases, including Alzheimer’s disease (AD)^17,18^. It has been demonstrated that microtubule defects in axons cause defective axonal transport^19^. Furthermore, memory loss could be attributed to microtubule depolymerization^20^. Moreover, Hwang et al. found that T2DM patients significantly alter the expression of proteins involved in the cytoskeleton’s structure, like the Z-disk component alpha actinin-2 and actin capping^21^.

Despite the known links between the cytoskeleton and age-related diseases, there are still multiple questions regarding the role of cytoskeleton disruption in the pathology of the diseases. On this matter, the cytoskeleton is considered one of the future research challenges because of its potential role in therapeutic strategies related to aging. In this study, an integrated workflow of machine learning models and differential expression analysis was utilized to investigate the effect of transcriptional dysregulation of cytoskeleton-associated genes in the pathology of Aging-related diseases, including Hypertrophic Cardiomyopathy (HCM), Coronary Artery Disease (CAD), Alzheimer Disease (AD), Idiopathic Dilated Cardiomyopathy (IDCM), and Type 2 Diabetes Mellitus (T2DM). Several cytoskeletons-related genes have been identified that accurately classify disease and control samples and were differentially expressed between patients and normal individuals. ARPC3, CDC42EP4, LRRC49, and MYH6 were identified for HCM. CSNK1A1, AKAP5, TOPORS, ACTBL2, and FNTA were identified for CAD. Moreover, ENC1, NEFM, ITPKB, PCP4, and CALB1 were identified for AD. Finally, two genes were identified for IDCM, MNS1, and MYOT, and one gene for T2DM, the ALDOB gene.

## Results

### Overview of the proposed model

**Fig. 1:**
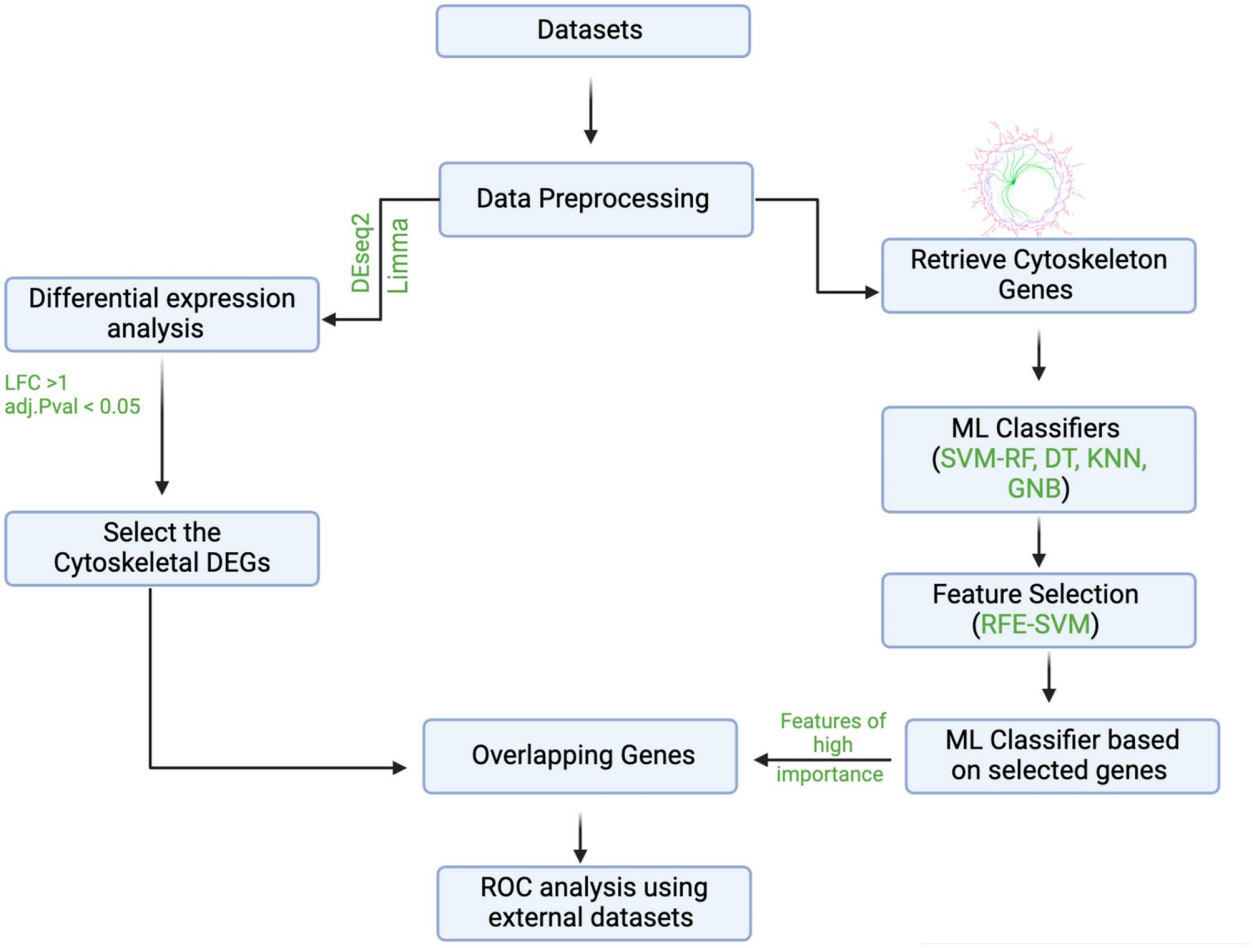
Schematic representation of the proposed workflow.

This study aimed to link the transcriptional dysregulation in cytoskeletal genes to age-related diseases, including HCM, CAD, IDCM, AD, and T2DM. The first step was to retrieve the cytoskeletal gene list from the Gene Ontology Browser (GO) with the ID (GO:0005856). The list contained 2304 genes (the complete list is provided in Supplementary Table S1). The list includes the main cytoskeleton components: microfilaments, intermediate filaments, microtubules, the microtrabecular lattice, and other structures characterized by a polymeric filamentous nature and long-range order within the cell. Multiple machine learning (ML) models were developed based on the cytoskeletal genes for each disease, where the Recursive Feature Elimination (RFE) technique was utilized to identify an informative set of genes. Moreover, differential expression analysis was also conducted for each disease, and we further focused on identifying the overlapping cytoskeletal genes between the RFE-selected features and the differentially expressed genes. The performance of the identified candidates was further validated using the Receiver Operating Characteristic (ROC) analysis on external datasets (Fig. 1).

Transcriptome data were retrieved for all the analyzed diseases (Table 1). Two different datasets were used for HCM to increase the number of control samples. The batch effect correction and normalization were done using the Limma Package^22^.

**Table 1:** The datasets analyzed in this study. For each disease, the GEO accession ID, the number of patients and control samples, and the number of cytogenes included are indicated.

Disease	GEO Accession	Patient	Control	Cytogenes
HCM	GSE32453	8	5	1696
HCM	GSE36961	106	39	1696
CAD	GSE113079	93	48	1989
AD	GSE5281	87	74	1561
IDCM	GSE57338	82	136	2167
T2DM	GSE164416	39	18	2188

### Support vector machines outperform the other four classifiers

Five different algorithms: Decision tree (DTs)^23^, Random Forest (RF)^24^, k-nearest Neighbors algorithm (k-NN)^25^, Gaussian Naive Bayes (GNN)^26^, and Support vector machines (SVMs)^27^ were utilized to build multiple classification models based on the normalized expression values of the cytoskeleton genes for each age-related disease separately. The Five-fold cross-validation (CV) method was utilized to assess the accuracy of each model. Table 2 shows that SVMs had the highest accuracy for all the diseases. The SVM classifier is well-suited for gene expression data due to its ability to handle large feature spaces and datasets and identify outliers^28^. This aligns with a previous study that used SVM to detect delicate patterns for complex diseases^29^. Therefore, SVM-based models were utilized in the subsequent analyses.

**Table 2:** The fivefold cross-validated accuracy for the following machine learning algorithms: DTs, RF, SVMs, and GNB.

Disease	DTs	RF	KNN	SVMs	GNB
HCM	89.15%	91.04%	92.33%	**94.85%**	82.17%
CAD	87.90%	92.21%	91.50%	**95.07%**	90.07%
AD	74.56%	83.23%	84.48%	**87.70%**	82.61%
IDCM	87.632%	94.048%	94.93%	**96.31%**	81.75%
T2DM	61.81%	80.75%	70.30%	**89.54%**	80.75%

### RFE-SVM selected a small subset of cytoskeletal genes that discriminate between patients and normal samples

**Fig. 2:**
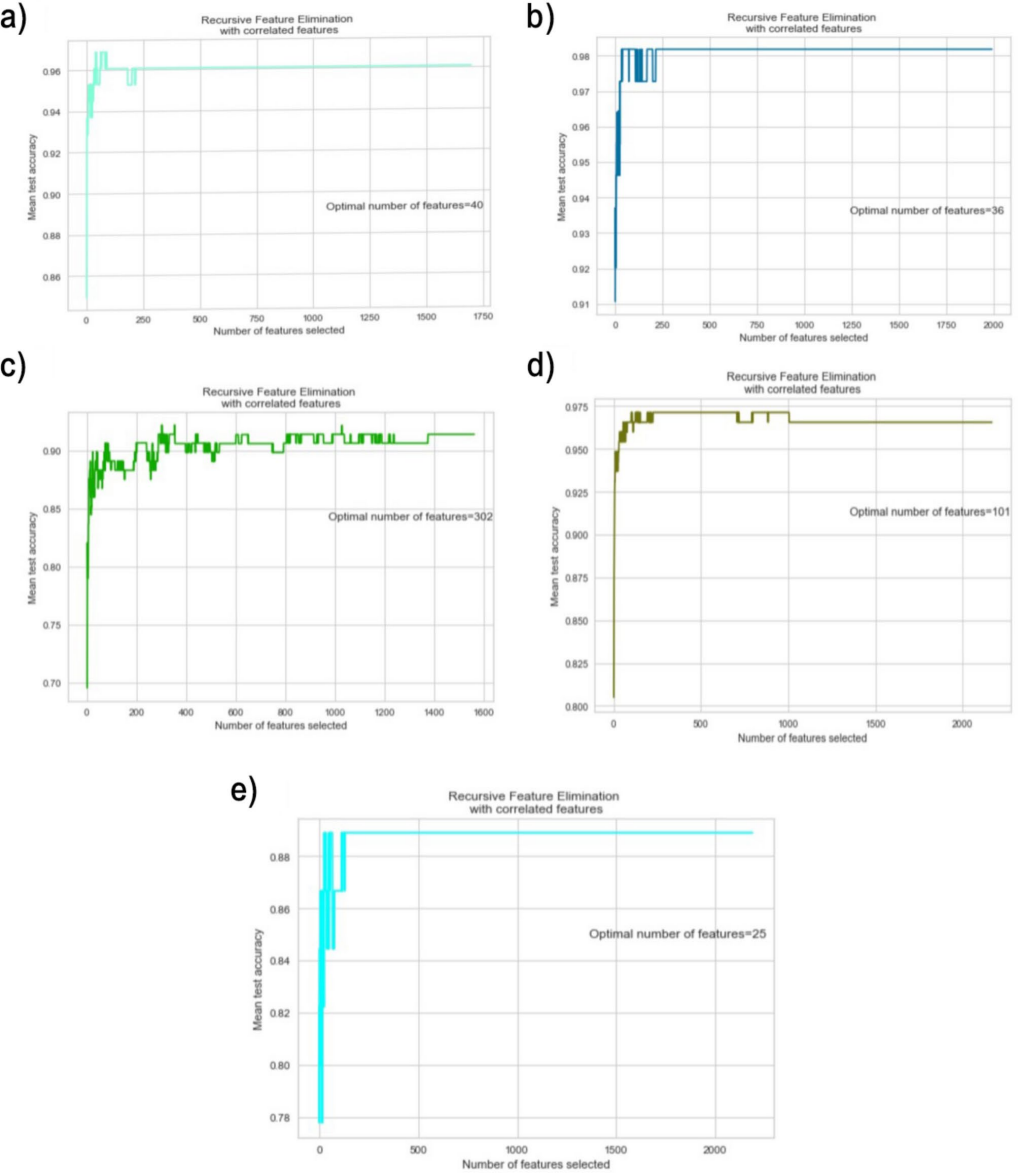
The incremental feature selection curves for RFE-based features and the achieved test accuracy for each age-related disease. (**a**) HCM, (**b**) CAD, (**c**) AD, (**d**) T2DM, (**e**) IDCM.

**Fig. 3:**
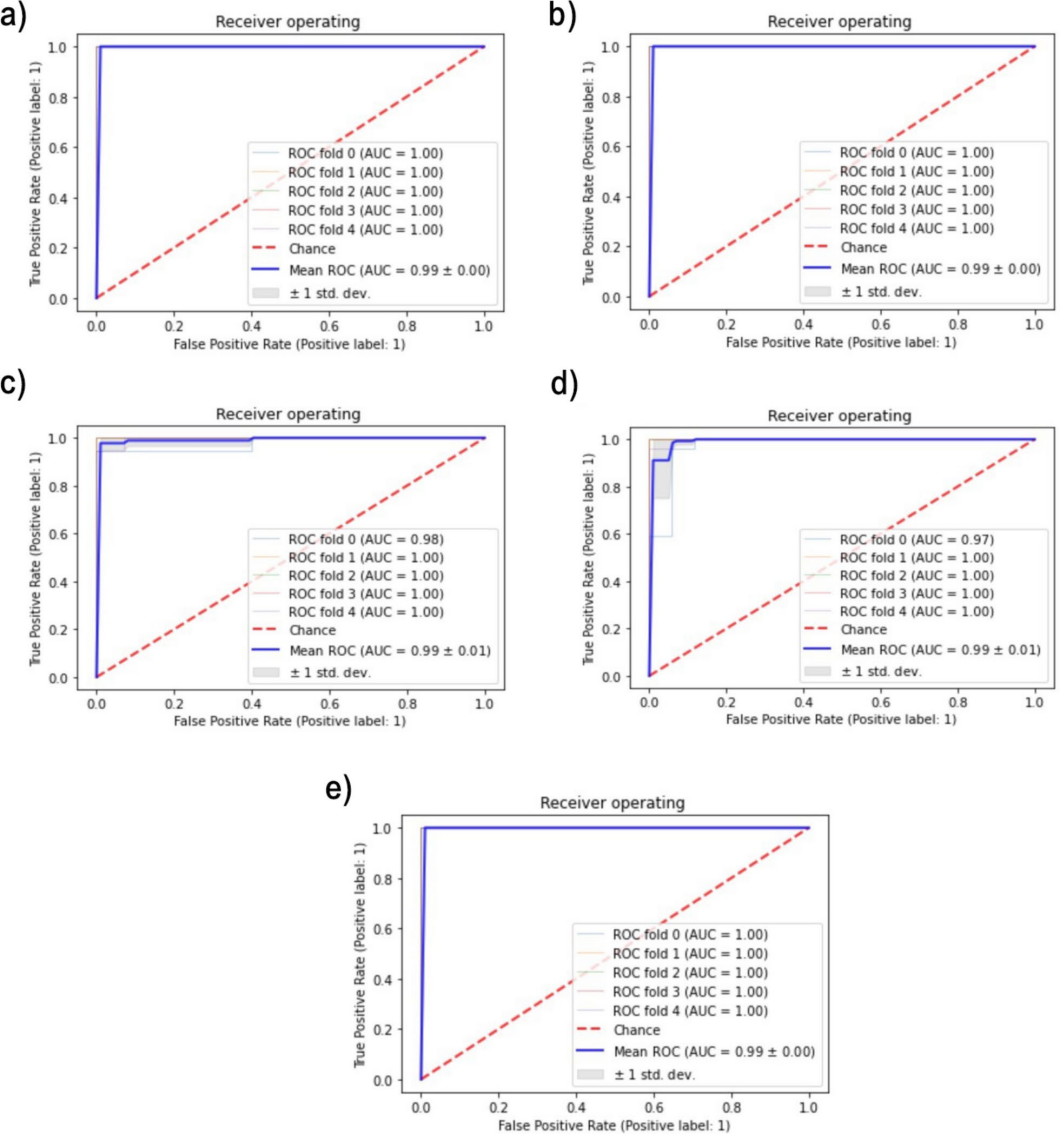
Average ROC curves of age-related disease models estimated using five-fold cross-validation and RFE-identified genes for each disease. (**a**) HCM, (**b**) CAD, (**c**) AD, (**d**) IDCM, (**d**) T2DM.

Recursive Feature elimination (RFE) was utilized to select the top gene signatures that differentiate patients from the normal samples in each disease. RFE is a wrapper feature selection method that recursively removes features with a definite step, then builds the model with the remaining features and calculates the accuracy^30^. RFE was employed along with the SVM classifier. After performing multiple iterations based on the number of cytoskeleton genes in each disease, the best subset of features is determined, starting with one feature, as RFE is more accurate with small steps^31^. Five-fold CV scores were calculated to evaluate the predictive performance of the selected features. The number of RFE selected features for each disease and their mean CV accuracy are shown in Fig. 2. The selected features were then utilized to train an SVM classifier for each disease. Table 3 illustrates a detailed evaluation in terms of accuracy, F1-score, recall, precision, and balanced accuracy. The Receiver Operating Characteristic (ROC) metric has also been calculated. Other metrics have also been calculated. This includes True Positives (TP), True Negatives (TN), False Positives (FP), False Negatives (FN), Positive Predictive Value (PPV), and Negative Predictive Value (NPV). Table 4 outlines the metric results for each disease, where high PPV values were observed across conditions, indicating strong reliability in positive predictions. Figure 3 illustrates the ROC analysis with the calculated area under the curve (AUC) values using stratified fivefold cross-validation. Two additional FS techniques, LASSO and ANOVA, were also investigated along with the SVMs-based model to identify the most relevant genes. Based on the F1-score performance, RFE paired with the SVC model outperformed for HCM, CAD, AD, and T2DM. However, LASSO achieved a slightly higher F1-score of 98.14% compared to RFE’s 97.47% for IDC (see Supplementary File 1).

**Table 3:** Performance of the SVM model based on the RFE selected features for all diseases.

Disease	Number of Features	Accuracy	Mean AUC	F1 Score	Recall	Precision	Specificity	Balanced Accuracy
HCM	40	96.77%	100%	92.15%	87.77%	100%	88%	90%
CAD	36	97.88%	100%	98.31%	97.77%	99%	97.77%	96.4%
AD	302	97.51%	99.4%	97.8%	96.88%	98.88%	98.88%	91.66%
IDCM	101	96.78%	99.4%	97.47%	99.25%	95.83%	99.25%	86.95%
T2DM	25	98.33%	100%	98.82%	100%	97.77%	100%	100%

**Table 4:** Performance of the SVM model on all diseases utilizing confusion matrix measures in addition to PPV and NPV metrics.

Disease	TP	TN	FP	FN	PPV	NPV
HCM	15	15	3	0	100%	83.3%
CAD	13	15	0	1	93%	100%
AD	22	8	2	0	100%	92%
IDCM	17	21	0	6	78%	100%
T2DM	5	7	0	0	100%	100%

### The overlapping cytoskeletal genes between all age-related diseases

After using RFE to find each disease’s most discriminative cytoskeleton genes, informative features overlapped across the diseases were identified. No overlapping was found between all the diseases. However, common genes between at least two diseases were identified (Supplementary Fig. S1). For example, the ANXA2 gene was common between AD, IDCM, and T2DM, while the TPM3 gene was common between AD, CAD, and T2DM. In addition, the SPTBN1 gene was common between AD, CAD, and HCM. Moreover, three genes were shared between AD and T2DM: MAP1B, RRAGD, and RPS3. On the other hand, three different genes were shared between AD and CAD: JAKMIP1, ABLIM3, and PDE4B. Furthermore, there were 20 overlapping genes between AD and IDCM (Table 5).

**Table 5:** Common genes selected by RFE between age-related diseases.

Diseases	Gene	Diseases	Genes
AD, IDCM, T2DM	ANXA2	AD , IDCM	DHCR24
AD, HCM, T2DM	TPM3		ANKRA2
AD, CAD, HCM	SPTBN1		TBL1X
AD, T2DM	MAP1B		SVBP
	RRAGD		COBL
	RPS3		TUBB2B
AD, CAD	JAKMIP1		FAM107A
	ABLIM3		ENC1
	PDE4B		IFT57
AD, HCM	RAB11FIP5		CFL1
	KLHL21		PTPN4
	BBS9		SEPTIN4
	PDLIM1		SYNM
	ARPC3		GPER1
HCM, T2DM	TUBA4A		ANK1
CAD, IDCM	FES		ATF4
	NOSTRIN		CEP19
CAD, HCM	LMNA		NR3C1
	NEXN		FAM161B
HCM, IDCM	MYL4		MAP2k6
	JUP		
	CORIN		

### Differential expression analysis (DEA) shows consistency with RFE for multiple genes and reveals common cytoskeletal gene signatures

DESeq2 for the T2DM dataset and Limma package for HCM, AD, CAD, and IDCM datasets were employed to identify the differentially expressed genes (DEGs) between the patients’ and normal samples for each disease. We then further focused on cytoskeleton genes for comparison. The thresholds for DEA were chosen to be adjusted *p*-value < 0.05 and |log2FC|> 1. For HCM, there were 86 differentially expressed genes, out of which 14 were cytoskeletal-related genes (Supplementary Table S2). For CAD, 243 genes were differentially expressed, and 25 were cytoskeleton genes (Supplementary Table S3). In the AD data, there were 489 DEGs, and 75 were cytoskeleton genes (Supplementary Table S4). In the IDCM disease, there were 92 DEGs, 6 of which were cytoskeletal genes (Supplementary Table S5). For T2DM, there were 624 DEGs, 56 of which were cytoskeletal genes (Supplementary Table S6). All cytoskeleton-related DEGs (CytoDEGs) are summarized in Table 6.

**Table 6:** The number of differentially expressed cytoskeletal genes for each disease.

Disease	CytoDEGS	Upregulated	Downregulated
HCM	14	6	8
CAD	25	6	19
AD	75	16	59
IDCM	6	3	3
T2DM	56	54	2

We focused on the overlapping cytoskeletal genes from DEA and RFE because genes may be differentially expressed yet not selected in Recursive Feature Elimination (RFE) due to differences in the criteria each method uses to assess relevance. Differential Expression Analysis finds genes with statistically significant expression differences between groups, such as normal versus disease states, but it does not measure a gene’s predictive value. On the other hand, a gene can exhibit notable changes in expression but lacks the reliability needed to differentiate disease samples in a predictive model effectively. Moreover, RFE chooses features based on their contribution to the model’s performance, assessing the added value of each gene alongside others in classifying or predicting disease^32^. In summary, there were four overlapping genes for HCM, five for CAD, 23 for AD, two for IDCM, and one for T2DM (Supplementary Fig. S2).

## Validation of cytoskeletal gene selection through ROC analysis

The ROC analysis was conducted to evaluate the performance of the identified common genes in differentiating between normal and disease conditions. The AUC value was computed for each gene to assess its discriminatory power between the disease and normal samples. This analysis was done across the primary and external datasets retrieved for validation (Supplementary Table S7). The description of the external datasets is included in Table 7. The ROC analysis on the external datasets yielded similar results, with AUC values for most genes consistent with those observed in the primary datasets.

**Table 7:** The external datasets utilized for validation. For each disease, the GEO accession ID, the number of control and patient samples.

Disease	GEO accession	Control	Patient
HCM	GSE180313	7	13
CAD	GSE20681	99	99
AD	GSE28146	7	8
IDCM	GSE141910	166	166
T2DM	GSE76895	32	36

**Fig. 4:**
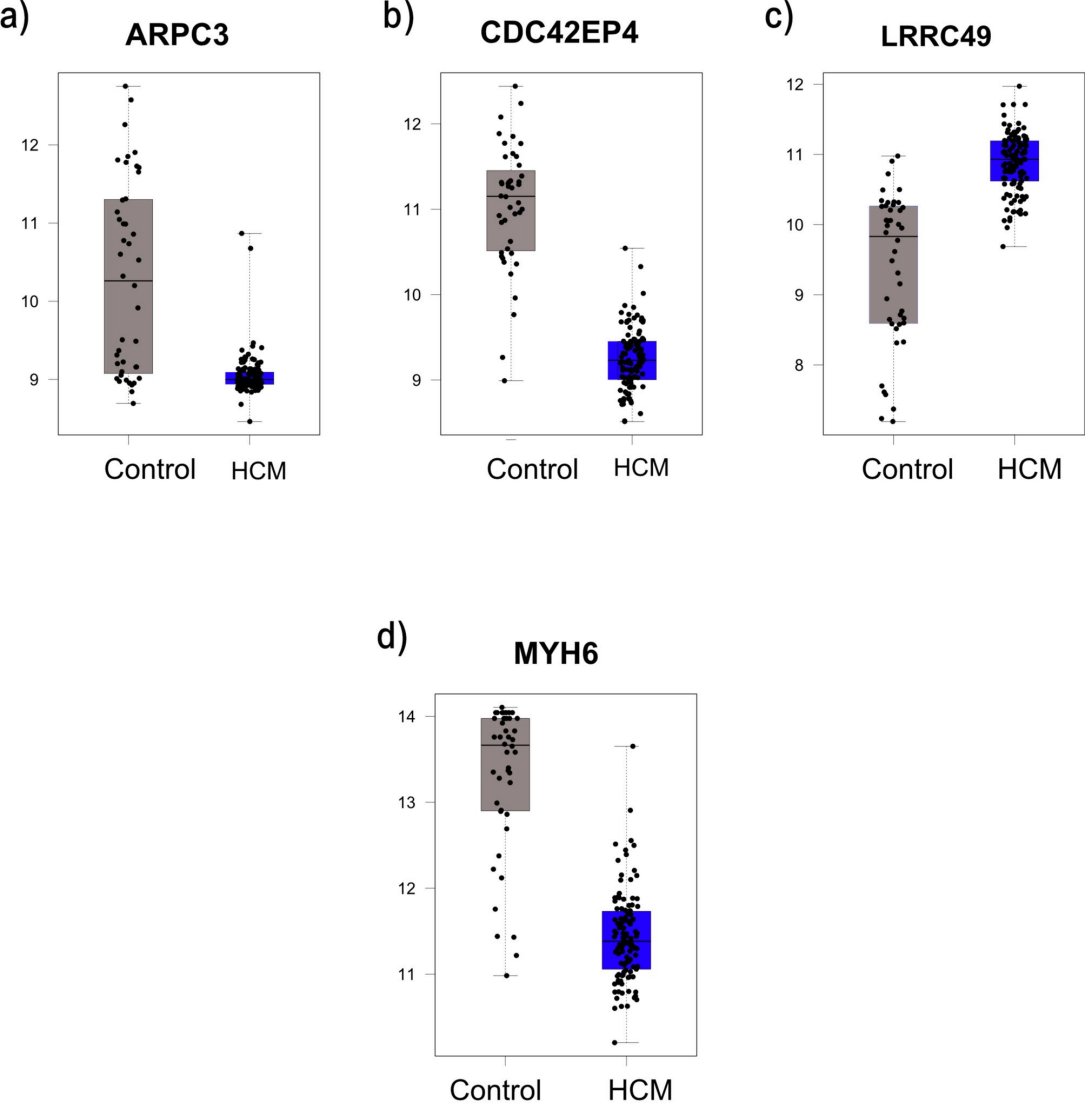
Boxplots representing the expression level of the common cytoskeletal-related genes in HCM.

**Fig. 5:**
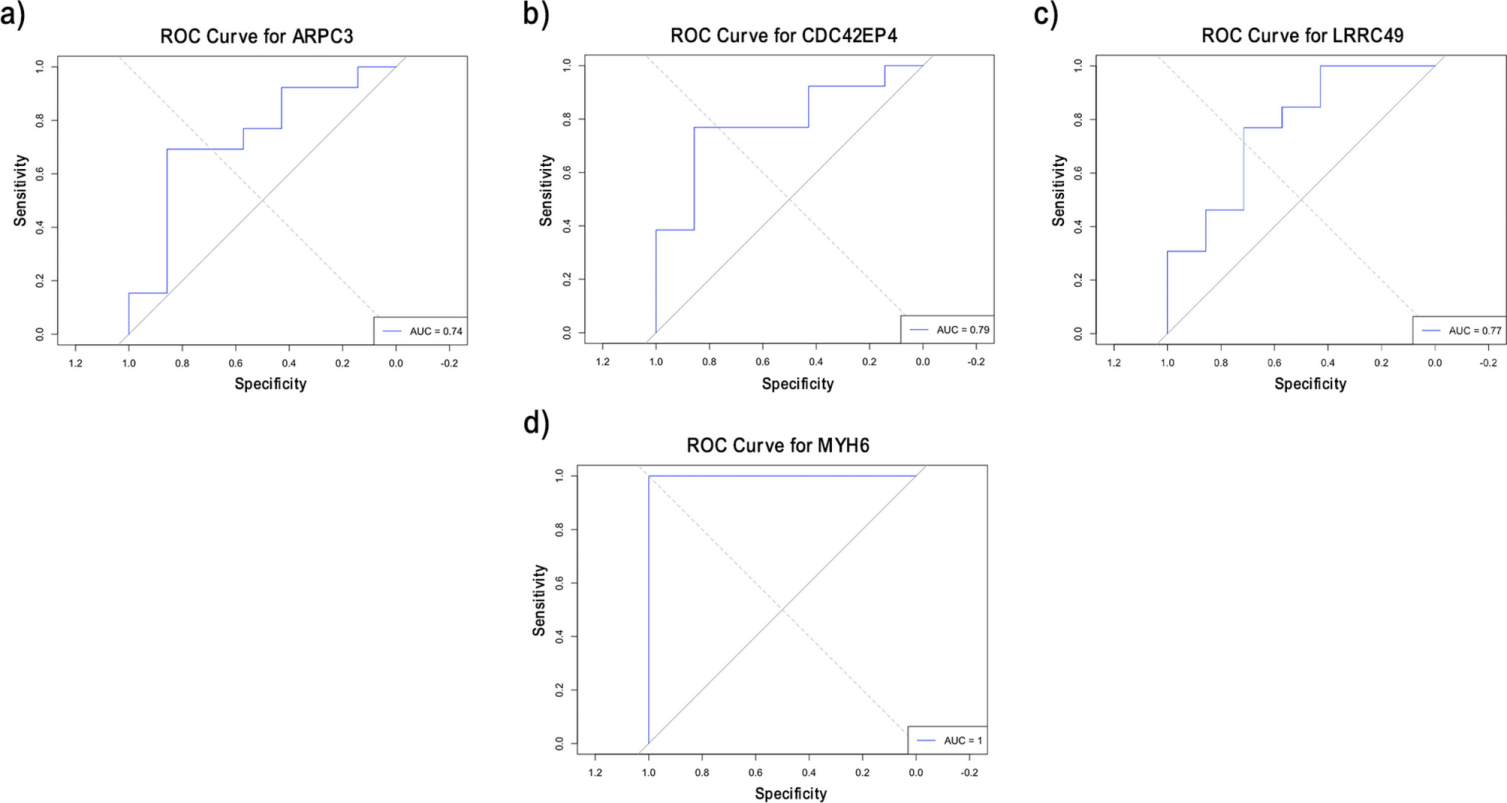
ROC analysis of the overlapping genes for HCM on the GSE180313 dataset.

For HCM, ARPC3, CDC42EP4, and MYH6 were downregulated in HCM patients, while LRRC49 was upregulated. All the overlapping genes play a role in actin-related pathways^33,34^(Fig. 4a-d). In addition, they achieved AUC values $\ge 0.74$ on the external dataset, especially for MYH6, which has an AUC of 1, highlighting its potential as a biomarker for HCM (Fig. 5 a-d).

**Fig. 6:**
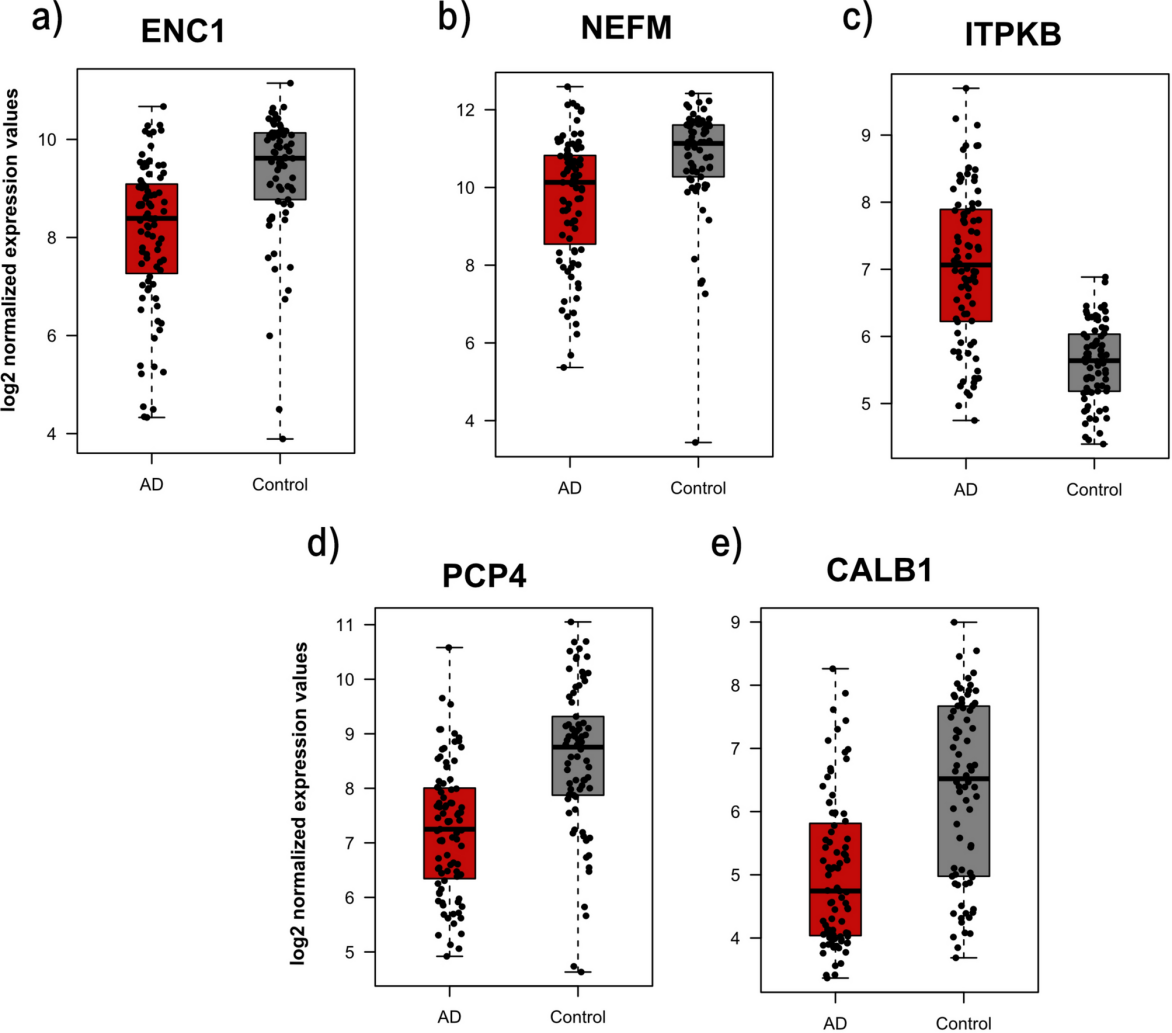
Boxplots representing the expression level of the overlapping genes between RFE and DEA for AD. The depicted genes have the highest AUC values based on the external validation.

**Fig. 7:**
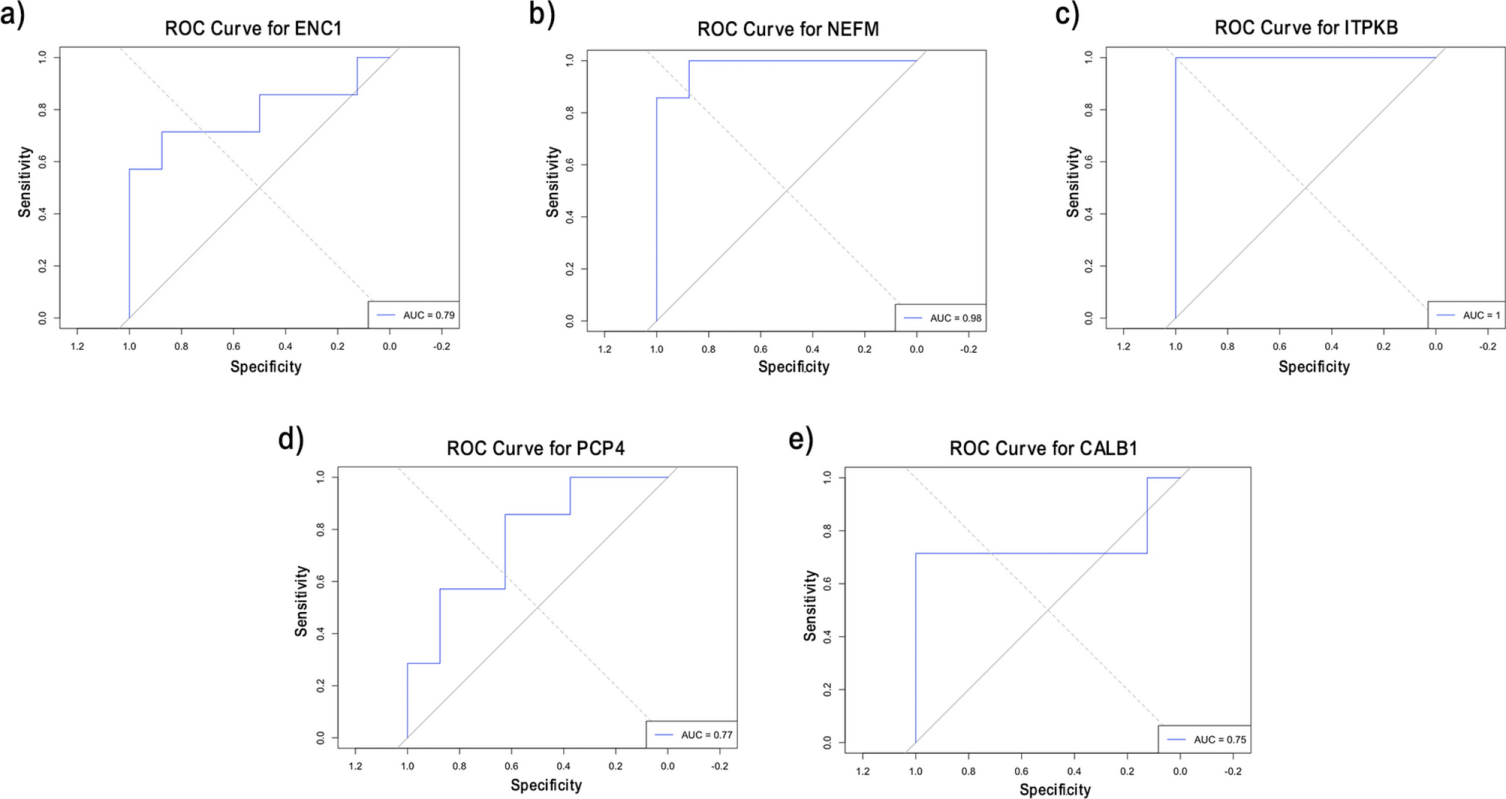
AUC values of the overlapping genes for AD on the external dataset GSE28146.

There were 23 identified common genes for AD. Genes with AUC > 0.75 are highlighted. All five genes were downregulated (Fig. 6. a-e) except for ITPKB. Interestingly, it also has an AUC value of 1 from the external dataset, which highlights its power in discriminating between AD patients and controls (Fig. 7. a-e).

**Fig. 8:**
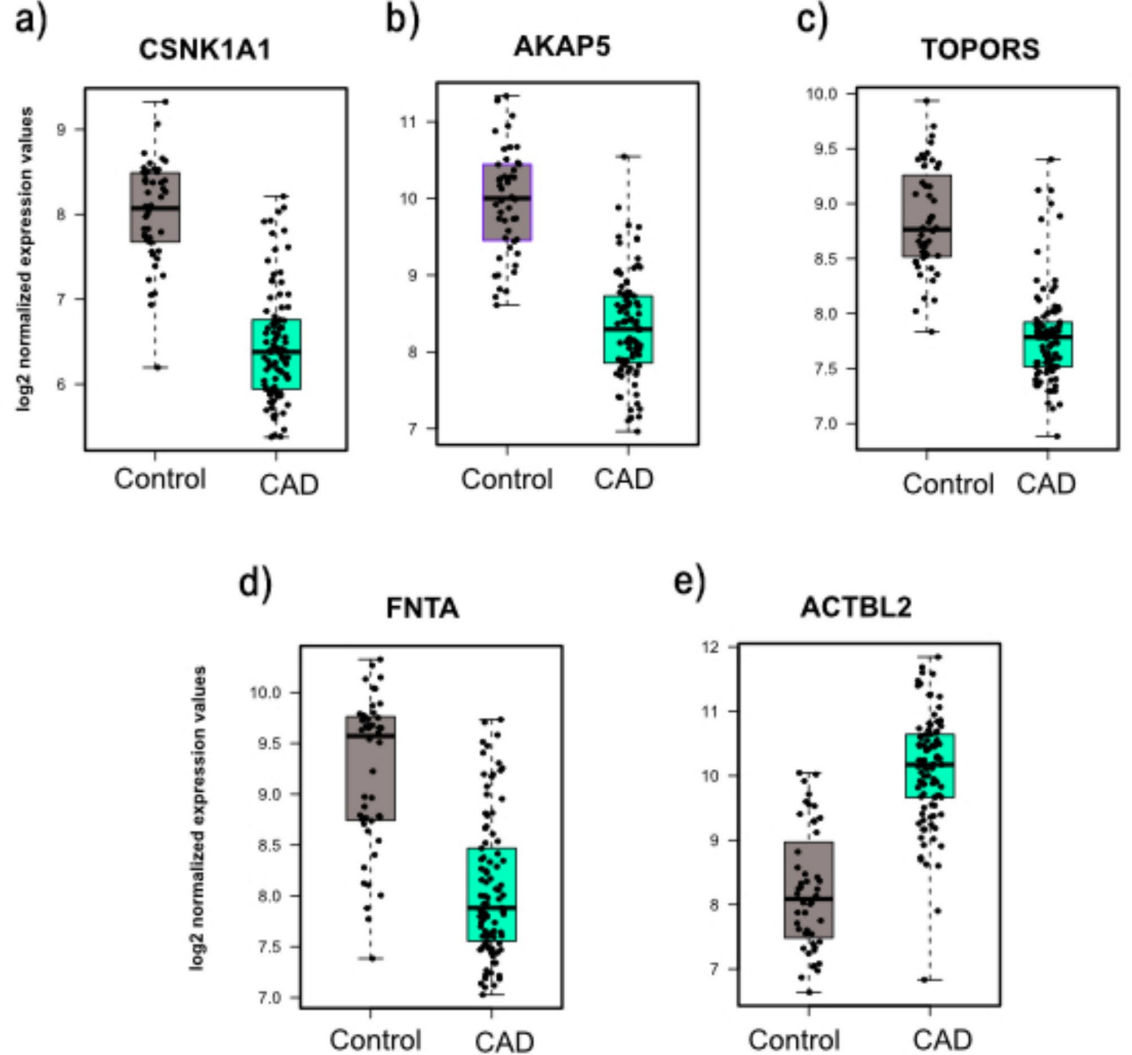
Boxplots representing the expression level of the overlapping genes between RFE and DEA for CAD.

**Fig. 9:**
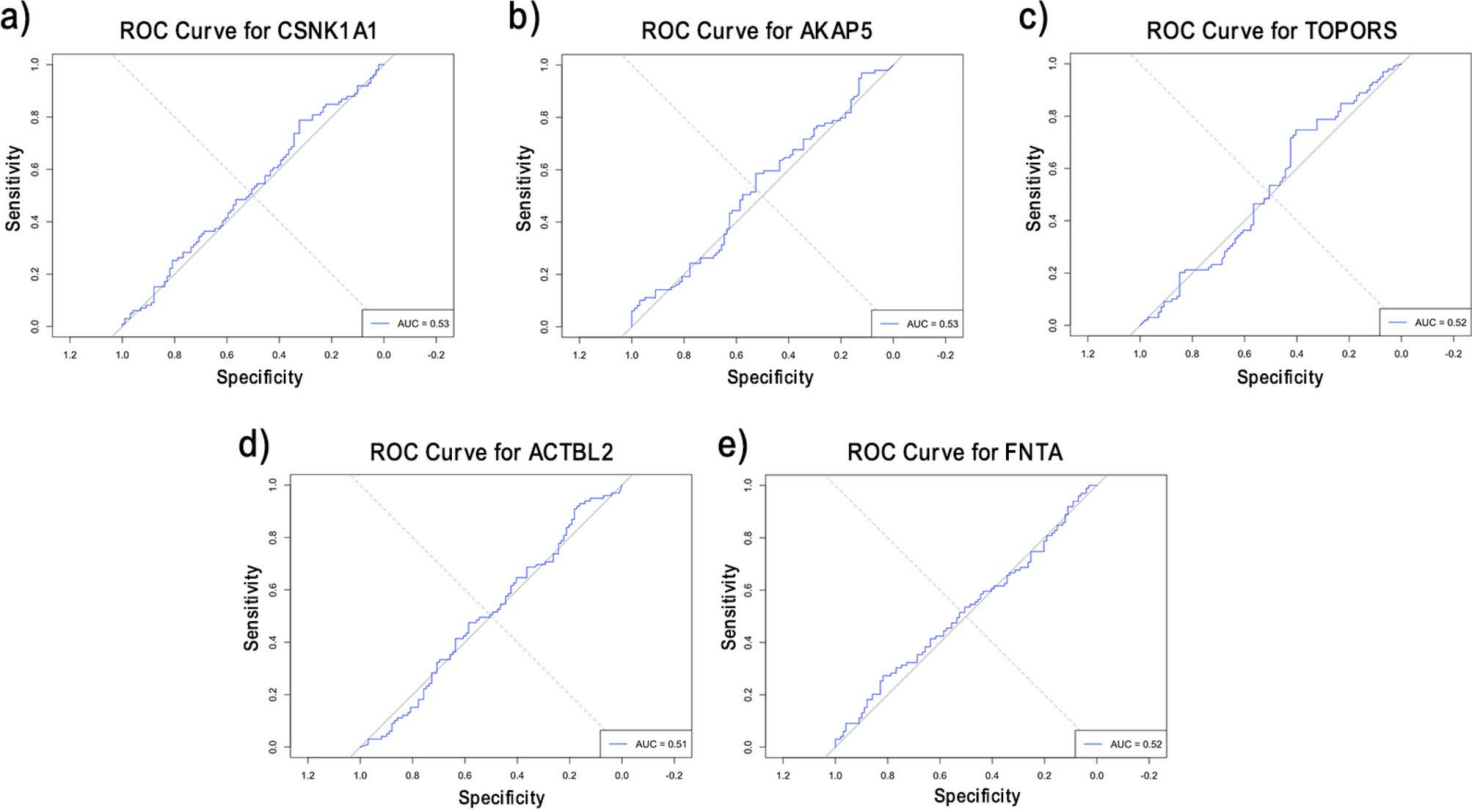
ROC analysis for the overlapping genes for CAD on the validation dataset (GSE20681).

Moreover, there were five overlapping genes for CAD: CSNK1A1, AKAP5, TOPORS, ACTBL2, and FNTA. All the genes were downregulated except for the ACTBL2 gene (Fig. 8 a-e). ROC analysis was done for the five overlapping genes in both primary and external datasets. For the primary dataset, all the identified genes had AUC values above 0.85, which explains their performance in differentiating between CAD patients and controls (Supplementary Table S7). However, for the external datasets, the same set of genes achieved AUC values above 0.5 (Fig. 9 a-e). This suggests that while the genes selected in the training phase effectively distinguished disease and normal conditions in Peripheral Blood Mononuclear Cells (PBMs), their performance may not generalize as well to whole blood cells. This could be explained by the difference in cell composition between them; PBMs consist of homogenous sets of immune cells that may provide distinct separation between normal and disease conditions, while whole blood cells are a heterogeneous mixture of different cell types^35^.

**Fig. 10:**
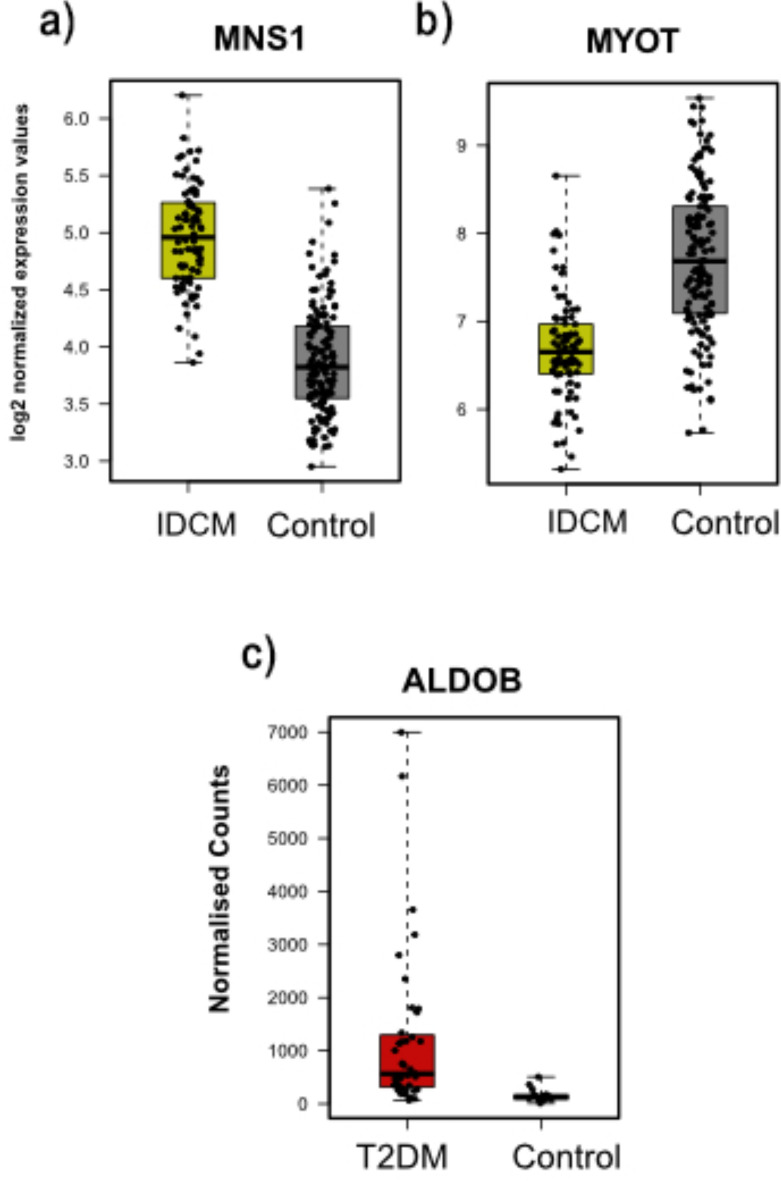
Boxplots representing the expression level of the common cytoskeletal-related genes in IDCM and T2DM.

**Fig. 11:**
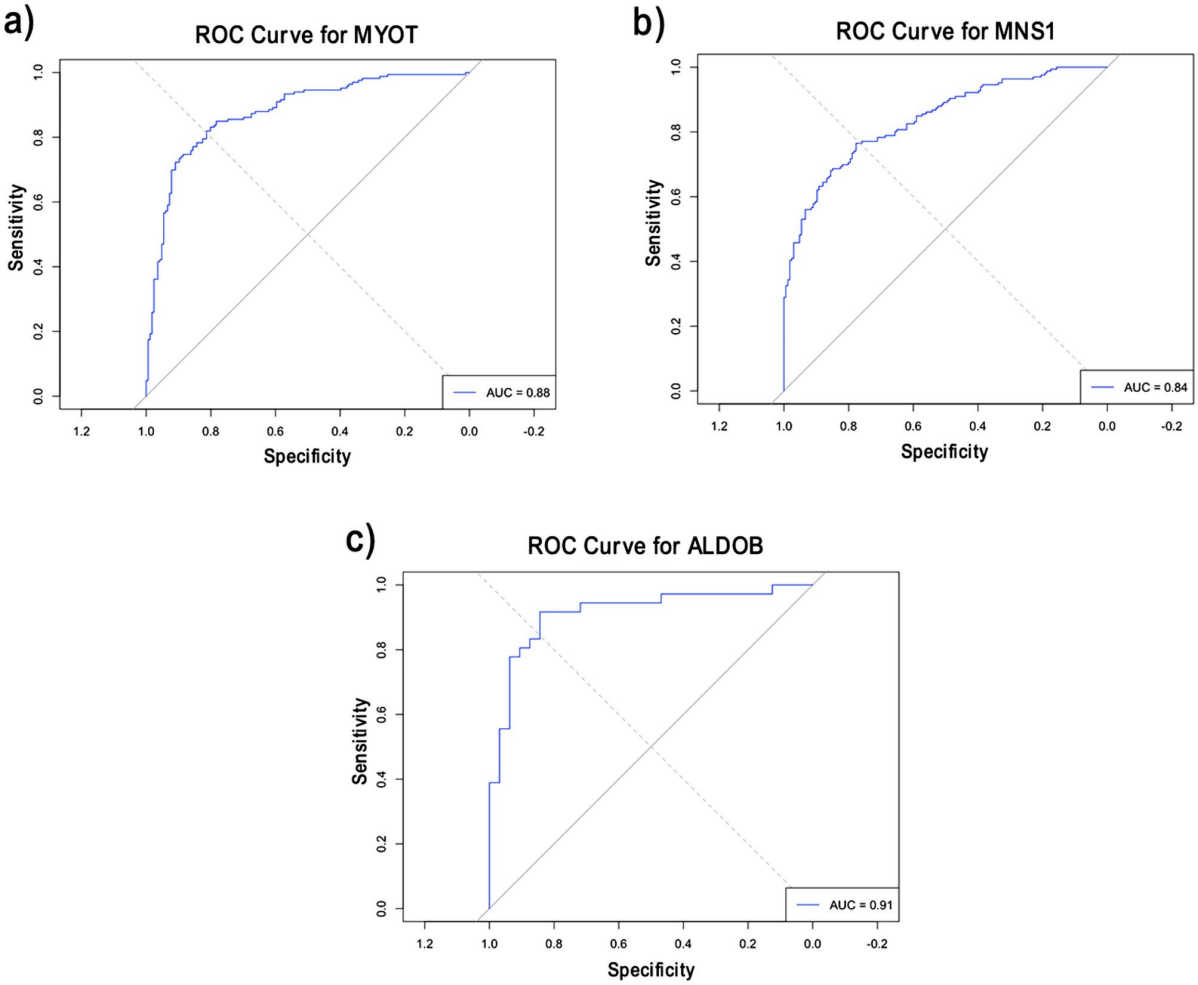
AUC values of the common genes for IDCM and T2DM on the external datasets.

Finally, IDCM had two overlapping genes: one upregulated gene, MNS1, and one downregulated gene, MYOT (Fig. 10 a-b). ROC analysis was also done for both genes for training and validation datasets. They had AUC values above 0.84 for both datasets, which reflects the role of these genes in the pathogenesis of IDCM disease (Fig. 11, a-b). The ALDOB gene was the only common gene for T2DM, and it was upregulated (Fig. 10c). It had an AUC value of 0.91 for the external dataset, which suggests the discrimination power for this gene in T2DM and control samples (Fig. 11c).

## Discussion

The cytoskeleton encompasses polymers of filamentous proteins that form an organized structure and affect different aspects of cell life^36^. It conveys the mechanical structure, allows a spatial organization, controls inter and intracellular transport, contributes to cell division, and participates in cell transduction^6–9^. A growing body of evidence implicates the role of the cytoskeleton in regulating cellular senescence, organismal aging, and neurodegeneration^37^. In the present study, we utilized an integrative approach from machine learning and differential expression analysis to identify potential biomarkers from the cytoskeleton genes for five age-related diseases, including Hypertrophic cardiomyopathy (HCM), coronary artery disease (CAD), Alzheimer’s Disease (AD), Idiopathic Cardiomyopathy (IDC), and Type2 Diabetes (T2DM). Differential expression analysis results in hundreds of statistically significant genes. Therefore, using all DEGs to develop diagnostic and prognostic prediction tools is impractical. However, machine learning and feature selection approaches identify a small set of marker genes^32^. Therefore, using an integrated workflow from both will help us determine a small group of genes that could potentially act as diagnostic biomarkers. Five machine learning algorithms, RF, DTs, KNN, GNB, and SVC, were initially utilized to build a classification model based on the normalized expression values of cytoskeletal genes for each disease separately. This step shows that SVC outperformed the classification of disease and normal classes compared to the other algorithms. SVMs are generally considered less prone to class imbalance issues since class boundaries are determined primarily by a limited number of support vectors. Therefore, the dataset size for each class may not significantly impact this classification method^38^. Afterward, three feature selection methods, RFE, LASSO, and ANOVA, were applied to remove useless or redundant features.

RFE, combined with the SVC model, consistently had higher scores. As a result, RFE feature selection was applied to remove the redundant features. The removal of the non-informative genes improves the accuracy of the model. Moreover, we ensured the significance of the selected features using differential expression analysis to identify the transcriptional changes between samples of the five age-related diseases and their normal samples. Finally, ROC analysis was performed for each disease separately using external datasets to validate the performance of the overlapping genes. In line with our hypothesis, RFE-selected features overlapped with those dysregulated genes obtained from differential expression analysis (DEA). Below, we will discuss interesting links between some of the identified cytoskeletal genes and each disease to support the reliability of our prediction models and analysis.

For Hypertrophic Cardiomyopathy (HCM), four DEGs were also identified as RFE selected features. ARPC3, CDC42EP4, LRRC49, and MYH6 were downregulated in HCM patients, and LRRC49 was upregulated (Fig. 4a-d). LRRC49 and MYH6 were the most dysregulated genes in our analysis. MYH6 is a gene that encodes many isoforms of myosin in adult hearts with its homolog MYH7, which explains the possible association with HCM^39^. MYH6 and ARPC3 may interact genetically through their roles in cytoskeletal dynamics. For instance, it has been reported that a rare missense mutation in the motor domain of β-cardiac myosin, encoded by the MYH7 gene, is associated with impaired actin filament motility in HCM^40^. LRRC49 is one of the proteins containing Leucine‐rich repeats (LRRs) that act as versatile structural domains in protein–protein interactions^41^. To our knowledge, no reported link exists between LRRC49, AD, and cardiac functions. However, LRRC49 dynamic changes with other cytoskeleton proteins contribute to the secretion and processing of proinsulin^42^.

For Coronary Artery Disease (CAD), five genes intersected between RFE and DEA analyses: CSNK1A1, AKAP5, TOPORS, ACTBL2, and FNTA. All these genes were downregulated except for the ACTBL2 gene (Fig. 5a-e). ACTBL2, AKAP5, and CSNK1A1 were the most dysregulated genes with Log_2_FC > =|1.5|. ACTBL2-encoded protein is abundantly expressed in VSMCs during biochemical stress, essential in the vascular smooth muscle cells (VSMCs) migration response and formation of stress fibers^43,44^. Our result shows that ACTBL2 was upregulated in CAD, and intriguingly, previous proteomic analysis revealed that ACTBL2 was the top upregulated protein in the aorta of aged people^45^. AKAP5 controls cardiac normal function by regulating the activity of cardiac calmodulin kinase II (CaMKII) and calcineurin (CaN) and recycling of the β1-adrenergic receptors (*β1ARs*)^46,47^. CaMKII is activated in diabetes, significantly contributes to the risk of cardiac arrhythmias, and has been involved in insulin secretion alteration^48^. Furthermore, AKAP5 knockdown can enhance cardiac dysfunction and hypertrophy developed with age^49,50^. Moreover, The Microarray expression data showed that CSNKA1A was dysregulated in patients with familial hypercholesterolemia (FH) disorder, which induces atherosclerosis and cardiovascular disease development^51,52^.

The ROC Analysis for overlapping genes in AD revealed that several genes, including ENC1, NEFM, ITPKB, PCP4, and CALB1, performed well in both primary and validation datasets, with AUC values exceeding 0.75. Notably, ITPKB achieved an AUC of 1.0, demonstrating its strong discriminatory power, effectively distinguishing between AD and normal conditions. ITPKB is linked to various neurodegenerative diseases, including AD, due to its role in synaptic function. Additionally, studies have shown that ITPKB affects the inositol phosphate signaling pathway, essential for synaptic plasticity, neuronal survival, and memory formation. Recent research also indicates that ITPKB expression levels are significantly altered in the brain tissues of AD patients^53^.

For Idiopathic Dilated Cardiomyopathy (IDC), MNS1 and MYOT intersected between RFE and DEA and had high AUC. MYOT encodes a myotilin protein localized at the Z-disc^54,55^ and is essential in maintaining its structure and assembly by crosslinking actin filaments^56^. Interestingly, four nonsense mutations in MYOT were detected in patients suffering from myofibrillar myopathy^57^. MNS is known to be essential for cilia structure and function^58^. Previous studies have suggested a central role for cilia in cardiovascular diseases through its participation in fibrogenesis during myocardial injury^59^.

ALDOB was the only intersecting gene for Type 2 Diabetes (T2DM). Aldolase B isozyme is encoded by the ALDOB and expressed in the liver, kidney, and small intestine^60^. Aldolase B plays a prominent role in glycolysis and gluconeogenesis by catalyzing the cleavage of fructose-1-phosphate into glyceraldehyde and dihydroxyacetone phosphate^61^. Upregulation of Aldolase B in rats with metabolic syndrome contributed to the Methylglyoxal (MG) overproduction in vascular smooth muscle cells (VSMCs) and aorta^62^. Higher levels of MG have been implicated in the pathological events related to vascular disease in hypertension and diabetes^63–65^.

It is clear that modified expression of cytoskeletal genes is linked to age-related diseases. The identified genes could possibly act as novel biomarkers upon further wet lab validation. The overlap between the feature selection and differential expression data encourages the implementation of machine learning algorithms to develop models that predict diseases based on a low number of features. Hence, essential factors contributing to the early diagnosis and treatment of these incurable diseases can be accurately identified.

## Conclusion

Despite the extensive plethora of research efforts toward solving the complex nature of age-related diseases, many research efforts are still needed to find a therapy that can halt the escalation of symptoms and provide definitive treatments. Previous studies have shown that the modulation in the actin cytoskeleton integrity disrupts the function of somatic cells, stem cells, and gametes, leading to aberrant embryonic gametes. Therefore, approaches to maintain the cytoskeleton dynamics and integrity can offer potential means for therapy. This study investigated the transcriptomic changes in cytoskeletal genes in five age-related diseases, including HCM, CAD, AD, IDC, and T2DM, to find the association between the cytoskeleton genes and age-related diseases. Multiple machine-learning algorithms were used to identify each disease based only on cytoskeletal genes and their regulators. Our results showed that the Support Vector machines (SVM) algorithm performed the normal-disease classification with the highest accuracy.

Furthermore, the Recursive feature elimination (RFE) method was used to remove redundant features. Differential expression analysis (DEA) was also conducted, in which we highlighted the cytoskeletal genes identified by both feature selection and found to be overexpressed or downregulated. The findings propose a strong association between the dysregulation of cytoskeletal genes and age-related diseases and recommend that the identified genes be further validated in the wet lab to be used as biomarkers for the diseases.

## Methods

### Obtaining the complete list of cytoskeleton genes

The complete list of the annotated cytoskeleton genes was retrieved from the Gene Ontology Browser (GO) with the ID (GO: 0,005,856) in the Cellular Component category. The list contains 2302 genes, including intermediate filaments, microfilaments, microtubules, the microtrabecular lattice, and other structures characterized by a polymeric filamentous nature and long-range order within the cell.

### Data acquisition for primary analysis

All datasets used in this study are obtained from the National Center for Biotechnology Information (NCBI) Gene Expression Omnibus (GEO). Although there are many available public data for each disease, the first filtration step was to select datasets featuring the highest number of cytoskeletal genes. All the datasets included at least 65% of the total cytoskeletal genes.

#### Hypertrophic cardiomyopathy (HCM)

Two raw gene expression datasets, GSE32453 and GSE36961, were retrieved from GEO. The first dataset comprised eight HCM patients and five controls, while the second included 106 HCM patients and 39 controls. The first dataset samples originated from the cardiac septum, while the others were from myectomy Tissue. The platform used for both was the Illumina HumanHT-12 V3.0 expression bead chip.

#### Coronary artery disease (CAD)

Raw expression data from the GSE113079 dataset were downloaded. The expression data for peripheral blood samples included 93 patients and 48 controls. The platform used is Agilent-067406 Human CBC lncRNA + mRNA microarray V4.0. The mRNA data was only utilized.

#### Alzheimer’s disease (AD)

Raw CEL files with the accession number GSE5281 were utilized. The data included 87 patients and 74 controls. The samples were from different brain regions: the entorhinal cortex, hippocampus, medial temporal gyrus, posterior cingulate, superior frontal gyrus, and primary visual cortex. Laser capture microscopy eliminated tissue heterogeneity before the expression profiling in all brain regions.

#### Idiopathic dilated cardiomyopathy (IDCM)

Raw CEL files for IDCM expression data (GSE57338) were retrieved. The data contained samples from 136 controls and 82 IDCM patients. All samples were taken from the cardiac tissue. Affymetrix Human Gene 1.1 ST Array [transcript (gene) version] was utilized for the analysis.

#### Type 2 diabetes (T2DM)

The processed RNA seq data for T2DM was obtained with the accession number (GSE164416). The dataset included 133 samples from pancreatic islets of human donors, stratified into four groups based on their diabetes status: 18 were non-diabetic (ND), 41 had impaired glucose tolerance (IGT), 35 had Type 3c diabetes (T3cD), and 39 had Type 2 diabetes (T2D). Only samples for non-diabetic and Type 2 diabetes (T2D) were used.

### Data acquisition for external validation

External datasets obtained from the National Center for Biotechnology Information (NCBI) Gene Expression Omnibus (GEO) were retrieved for validation. Datasets featuring the identified common cytoskeletal genes were selected.

#### Hypertrophic cardiomyopathy (HCM)

GSE180313 comprised 13 HCM patients and seven controls. All Samples are myocardial samples. The platform used was the Illumina NovaSeq 6000 (Homo sapiens).

#### Coronary artery disease (CAD)

Raw expression data from the GSE20681 dataset was downloaded. The expression data for Whole blood cells isolated from patients before undergoing cardiac catheterization included 99 patients and 99 controls. Agilent-014850 Whole Human Genome Microarray 4 × 44K G4112F was utilized.

#### Alzheimer disease (AD)

Normalized gene expression data with the accession number GSE28146 was utilized. The samples were from hippocampal gray matter from formalin-fixed, paraffin-embedded (FFPE) hippocampal sections.

#### Idiopathic dilated cardiomyopathy (IDCM)

Pre-processed gene expression data files for DCM GSE141910 were retrieved. The data contained samples from non-failing healthy donors, as well as peripartum cardiomyopathy (PPCM), hypertrophic cardiomyopathy (HCM), and dilated cardiomyopathy (DCM) samples. All 166 non-failing healthy samples and 166 dilated cardiomyopathy were used for the validation. All samples were taken from Left ventricular tissue. Illumina HiSeq 2500 (Homo sapiens) was employed for the analysis.

#### Type 2 diabetes (T2DM)

The processed expression data for T2DM was obtained with the accession number GSE76895. The dataset includes 103 pancreatic islets for human donors, stratified into four groups based on their diabetes status: 32 were non-diabetic (ND),15 had impaired glucose tolerance (IGT), 20 had Type 3c diabetes (T3cD), and 36 had Type 2 diabetes (T2D). Only samples for non-diabetic and Type 2 diabetes (T2D) were used.

### Data pre-processing

All the pre-processing for the data was done using R (Version 4.1.1).

#### Hypertrophic cardiomyopathy (HCM)

The Limma package (version 3.50.3) was used for the processing step. The ReadTargets function was used to import the raw file in R studio. Then, the neqc function was utilized for background correction and normalization. Furthermore, the Avereps function was used to summarize the expression of multiple probes for the same genes. Finally, the removeBatchEffect function was used to ensure the datasets were homogeneous to eliminate the batch effect between them^22^.

#### Coronary artery disease (CAD)

The processing step was done using the Limma package (version 3.50.3). The read.maimages function was used to read the raw agilent files. The background Correct and normalizeBetweenArrays functions were executed for background correction and normalization, respectively^22^.

#### Alzheimer’s disease (AD)

Raw gene expression data from the Affy package (1.72.0) were utilized for the pre-processing step. The ReadAffy function was used to read the raw CEL files, and the RMA function was used for background correction and normalization^66^.

#### Idiopathic dilated cardiomyopathy (IDCM)

Oligo package was employed for the pre-processing. The read.celfiles function was used to read the raw CEL file with the pd.hugene11st.hs.entrezg package to annotate the probes^67^. Then, the RMA function was utilized to normalize and background correct the expression set.

#### Type 2 diabetes (T2DM)

The DEseq2 package (version 1.72.0) was used to convert the pre-processed data into a Deseq matrix, and org.Hs.eg.db (version 3.14.0) was utilized to convert ensemble ID into gene symbol^68^.

Each dataset normalized gene expression data was filtered to include the expression for the cytoskeletal genes only.

### Classification models

In the classification process, five different machine learning algorithms, including Decision Tree (DTs), Random Forest (RF), KNeighbors (KNN), Support vector machine (SVM), and GaussianNB (GNB) were implemented to build a classification model based on the cytoskeletal genes. Scikit-learn libraries were utilized^69^. Decision Trees (DTs) are supervised learning models that can be used for classification and regression problems^23^. The DTs algorithm was employed with the default parameters, with initial tree depth = 3 and gini impurity as a splitting measure. All parameters were also set to default in the RF model. Gini impurity was utilized to measure the quality of a split, and the minimum number of samples required to be at a leaf node was set to 2.

K Nearest Neighbours, or KNN, is a supervised learning model that utilizes the proximity method to assign a group to each point^25^. The number of neighboring samples to use for imputation was set to 5. GaussianNB (NB) is a probabilistic supervised machine learning algorithm based on the Bayes theorem used for classification. NB was employed with the default parameters; priors were none.

SVMs are a set of supervised learning methods used for classification and regression. SVM.SVC used parameters were set with kernel = “linear” as this is the default of SVMs. Five-fold cross-validation was used to evaluate the accuracy of the predicted models on the training and testing data using the cross_value_score function.

### Feature selection with recursive feature elimination technique

RFE is a wrapper feature selection method that selects the most relevant features for building an efficient predictive model for the target variable^30^. Multiple studies have demonstrated its robustness to data over-fitting compared to other FS techniques, improving model generalization in various fields such as genomics, proteomics, and metabolomics^70^. RFE with SVM classifier (RFE-SVM) as an estimator was implemented using Python. It initiates searching all the features in the training dataset by fitting the model, ranking all features by importance, removing the least relevant features, and re-fitting the model. Firstly, the RFECV function was used to determine the optimal number of features with fivefold cross-validation, and then the RFE function was used to select the features and fit the model.

### Tuning the parameters for the SVM models

Grid search with fivefold cross-validation was employed for hyperparameter optimization, which makes a complete search over a range of hyperparameter space for SVM.SVC algorithm^71^. The parameters were gamma = [1, 0.1, 0.01, 0.001, 0.0001], C = [0.1, 1, 10, 100, 1000], kernel = [“linear”,”rbf”]. This was done using the GridSearchCV function from the Scikit-learn Python library. Then, the tuned model was built using RFE-selected features. The first one is the Gamma value, which controls the distance of the single training point influence. The second parameter is the C value that optimizes the penalty for each misclassified point^72^. The Grid Search CV function was used to find the best parameters for each model. The C value for HCM, CAD, and AD was 10, while for IDCM and T2DM, it was 1 and 0.1, respectively. For the gamma parameter, the tuned value was 0.001 for HCM, CAD, AD, and IDA, while for T2DM, it was 1. The kernel type was “rbf” for AD, CAD, HCM, and IDC datasets and “linear” for T2DM.

### Differential expression analysis of microarray data (DEA)

The microarray data analysis was performed using the Limma package (version 3.50.3) for HCM, CAD, AD, and IDCM. The default analysis workflow was conducted on each dataset individually. The moderated t-statistic is used for significance analysis, where the standard errors are moderated across genes, i.e., embraced to a mutual value, utilizing a simple Bayesian model^73^. The results were filtered based on adjusted p-value < 0.05 and |log2 fold change|> 1. The complete list of DEGs was obtained before selecting the cytoskeleton genes.

### Differential expression analysis (DEA) of RNA-seq data

DEA for T2DM was conducted utilizing the Deseq2 package (version 1.72.0). The DESeq2 package evaluates the variance-mean dependence in the count data to identify the DEGs based on a negative binomial distribution. The differential analysis significance testing is based on a two-tailed Wald test^68^. The thresholds were based on adjusted p-value < 0.05 and |log2FC|> 1.

### Receiver operating characteristic (ROC) analysis

pROC R package (**Version 1.18.5**) was utilized to conduct the ROC analysis and evaluate the prediction potential of the identified cytoskeletal genes for each disease separately. ROC curves were generated on the training and validation datasets. AUC values were calculated and used as the performance metric.

## Supplementary Information

Supplementary Information 1.

Supplementary Information 2.

Supplementary Information 3.

## Data Availability

Publicly available datasets were analyzed in this study. All the datasets were derived from the public GEO data portal (https://www.ncbi.nlm.nih.gov/geo/).

## Abbreviations

HCM: Hypertrophic Cardiomyopathy
CAD: Coronary Artery Disease
AD: Alzheimer’s Disease
IDCM: Idiopathic Dilated Cardiomyopathy
T2DM: Type 2 Diabetes Mellitus
SVMs: Support Vector Machines
RFE: Recursive Feature Elimination
DTs: Decision trees
RF: Random forest
K-NN: K-Nearest Neighbors algorithm
GNN: Gaussian Naive Bayes
CV: Cross-validation
AUC: Area under the Curves
ROC: Receiver Operating Characteristic Curve
DEA: Differential expression analysis

## Code availability

The code for reproducing the transcriptomic analysis results will be available in the Github repository. See Code https://github.com/ComputationalBiologyLab/Cytoskeleton-/tree/main
