# Supplementary material for: A computational framework for identifying cytoskeletal genes associated with age-related diseases
Source: Sci Rep. 2025 Apr 26;15:14590. doi: 10.1038/s41598-025-97363-y (PMC12033331; doi:10.1038/s41598-025-97363-y)
Supplement: Supplementary file 2 — Supplementary Information 2. [file 41598_2025_97363_MOESM2_ESM.pdf]

A Computational Framework for Identifying Cytoskeletal Genes Associated with Age-Related Diseases

Reem A. Elghaish[1, 2], Nayera E. Attallah[1,2], Hesham Khaled [1,2], Asmaa S. Mekawy[1,2], Menattallah Elserafy[1,2,*] and Eman Badr[1,3,*]

[1] University of Science and Technology, Zewail City of Science and Technology, Giza, 12578, Egypt

[2] Center for Genomics, Helmy Institute for Medical Sciences, Zewail City of Science and Technology, Giza, 12578, Egypt

[3] Faculty of Computers and Artificial Intelligence, Cairo University, 12613, Giza, Egypt

[*]**Correspondence:** Eman Badr (emostafa@zewailcity.edu.eg) and Menattallah Elserafy (melserafy@zewailcity.edu.eg)

Comparative analysis for feature selection techniques

Two feature selection (FS) techniques have been explored in addition to the recursive feature elimination techniques to study their impact on the achieved performance. The Least Absolute Shrinkage and Selection Operator (LASSO) regression was used for feature selection. Lasso offers high accuracy for prediction models since the method is based on coefficient shrinkage, decreasing the variance and minimizing bias [1]. It optimizes the cost function to reduce the absolute values of the coefficients. Based on the coefficients, Lasso reduces redundant features and chooses only features with non-zero coefficients [2,3]. GridSearchCV function was used to optimize the alpha parameter for each dataset. The optimized alpha for Hypertrophic Cardiomyopathy (HCM), Coronary Artery Disease (CAD), Alzheimer's disease (AD), Idiopathic Dilated Cardiomyopathy (IDC), and Type 2 Diabetes Mellitus (T2DM) was 0.1. The number of features for HCM, CAD, AD, IDC, and T2DM were 11, 10, 19, 16, and 18, respectively. Table 1 illustrates a detailed evaluation in terms of accuracy, F1 score, recall, and specificity.

Table 1 Performance of the SVM model based on the lasso selected features for all diseases.

Disease	Accuracy	Mean AUC	F1 Score	Recall	Specificity
**HCM**	94.23 %	99.78 %	86.98 %	100 %	78.88 %
**CAD**	97.19 %	100 %	97.85%	98.09 %	97.77 %
**AD**	91.32 %	96.8 %	92.52%	91.12 %	94.31 %
**IDCM**	97.71 %	99.29 %	98.14%	98.51 %	97.77 %
**T2DM**	93.33 %	100%	95.56%	92.23 %	100 %

On the other hand, Analysis of Variance (ANOVA) is a filter feature selection method based on comparing the means for two or more samples. ANOVA F-test is also a class of statistical tests that evaluate the ratio between the variance of the samples [4,5]. The number of features was tuned to select the number with the highest accuracy. The optimized features were 67, 83, 70, 76, and 96 for HCM, CAD, AD, IDC, and T2DM, respectively. ANOVA f-test was implemented using the f_classif function from the scikitlearn library with the selected features. A detailed evaluation and scores are summarized in Table 2 in terms of accuracy, F1 score, recall, and specificity.

Table 2 Performance of the SVM model based on the ANOVA selected features for all diseases.

**Disease**	Accuracy	Mean AUC	F1 Score	Recall	Specificity
**HCM**	96.14%	100%	90.98%	100 %	85.55%
**CAD**	92.93%	99.66%	94.38%	97.27%	92.33%
**AD**	89.45%	95.72%	90.75%	88.68%	93.2%
**IDCM**	96.34%	98.39%	97.07%	96.42%	97.77%
**T2DM**	89.54%	98.125%	92.55%	91.55%	94.64%

Reference:

[1] Tibshirani, R. Regression shrinkage and selection via the lasso. *Journal of the Royal Statistical Society Series B: Statistical Methodology*, *58*(1), 267-288 (1996).

[2] Muthukrishnan, R., & Rohini, R. LASSO: A feature selection technique in predictive modeling for machine learning. In *2016 IEEE international conference on advances in computer applications (ICACA)* 18-20 (2016). doi:10.1109/ICACA.2016.7887916.

[3] Ghosh, P. et al. Efficient prediction of cardiovascular disease using machine learning algorithms with relief and LASSO feature selection techniques. *IEEE Access*, *9*, 19304-19326 (2021).

[4] AJPAS, A. A feature selection based on one-way-ANOVA for microarray data classification. *AJPAS JOURNAL*, *3*, 1-6 (2016).

[5] Dissanayake, K., & Md Johar, M. G. Comparative study on heart disease prediction using feature selection techniques on classification algorithms. *Applied Computational Intelligence and Soft Computing*, 1, 5581806 (2021).
