# Supplementary material for: A computational framework for identifying cytoskeletal genes associated with age-related diseases
Source: Sci Rep. 2025 Apr 26;15:14590. doi: 10.1038/s41598-025-97363-y (PMC12033331; doi:10.1038/s41598-025-97363-y)
Supplement: Supplementary file 3 — Supplementary Information 3. [file 41598_2025_97363_MOESM3_ESM.pdf]

A Computational Framework for Identifying Cytoskeletal Genes Associated with Age-Related Diseases

Reem A. Elghaish[1, 2], Nayera E. Attallah[1,2], Hesham Khaled [1,2], Asmaa S. Mekawy[1,2], Menattallah Elserafy[1,2,*] and Eman Badr[1,3,*]

[1] University of Science and Technology, Zewail City of Science and Technology, Giza, 12578, Egypt

[2] Center for Genomics, Helmy Institute for Medical Sciences, Zewail City of Science and Technology, Giza, 12578, Egypt

[3] Faculty of Computers and Artificial Intelligence, Cairo University, 12613, Giza, Egypt

[*]**Correspondence:** Eman Badr (emostafa@zewailcity.edu.eg) and Menattallah Elserafy (melserafy@zewailcity.edu.eg)

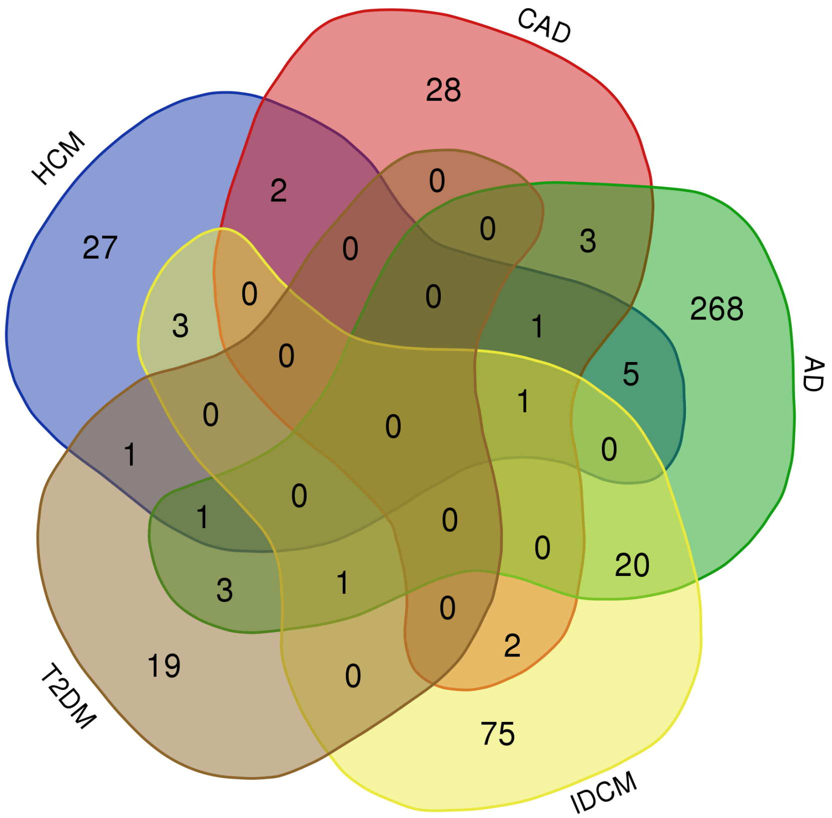

Supplementary Fig. 1 A Venn diagram illustrating the overlapping cytoskeletal genes between all age-related diseases based on the recursive feature elimination (RFE) features.

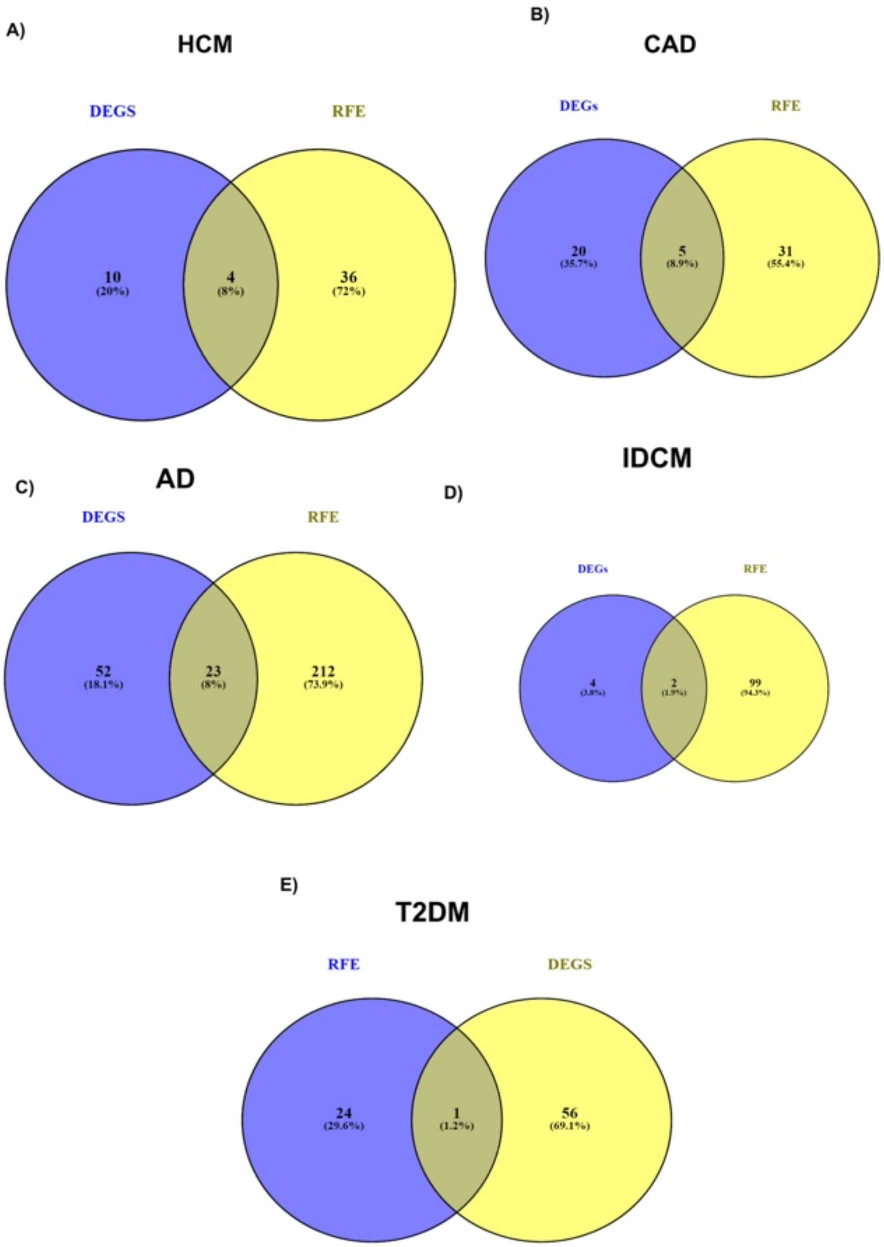

Supplementary Fig. 2 A Venn diagram shows the overlap between selected features utilizing the RFE technique and differentially expressed genes (DEGs) in all the diseases A: HCM, B: CAD, C: AD, D: IDCM, E: T2DM.
